# Three-dimensional finite element analysis of maxillary molar distalization treated with clear aligners combined with different traction methods

**DOI:** 10.1186/s40510-024-00546-y

**Published:** 2024-12-09

**Authors:** Hongyu Gao, Liangyu Luo, Jun Liu

**Affiliations:** State Key Laboratory of Oral Diseases & National Center for Stomatology & National Clinical Research Center for Oral Diseases, Department of Orthodontics, West China Hospital of Stomatology, Sichuan University, Chengdu, Sichuan 610041 China

**Keywords:** Finite element method, Clear aligner, Molar distalization

## Abstract

**Background:**

This study aimed to analyze the effects of maxillary molar distalization using clear aligners with different intramaxillary and intermaxillary traction via the three-dimensional (3D) finite element method.

**Methods:**

A 3D finite element model consisting of the maxilla, mandible, dentitions, periodontal ligaments (PDLs), attachments, and clear aligners was constructed. Five groups were established based on different traction modalities: group 1 (control group); group 2 (orthodontic mini-implants (OMIs) were implanted between the maxillary first molars and the second premolars on the buccal side); group 3 (OMIs were implanted in the infrazygomatic crest area between the maxillary first and second molars on the buccal side); group 4 (OMIs were implanted between the maxillary first molars and the second premolars on the palatal side); and group 5 (class II elastics were utilized between the maxillary canines and the mandibular first molars). OMIs were implanted 4 mm away from the alveolar crest in each experimental group. A force of 1.5 N was applied to each experimental group. The 3D displacement of the target teeth and stress distribution around the PDLs were analyzed.

**Results:**

Group 4 exhibited the least amount of torque change in the upper anterior teeth and the highest displacement of the maxillary second molars. Group 3 showed smaller changes in anterior teeth torque and higher molar distalization efficiency compared to group 2. Group 5 showed adverse effects such as anterior teeth extrusion and mandibular anchorage loss.

**Conclusion:**

OMIs implanted on the palatal side have advantages in preserving anterior teeth anchorage and improving the efficiency of molar distalization compared to those positioned on the buccal side. OMIs implanted in the infrazygomatic crest area between the first and second molars on the buccal side demonstrate benefits in the aforementioned aspects when compared to OMIs implanted between the first molars and the second premolars on the buccal side.

## Background

With advantages such as aesthetics, non-invasiveness and cleanliness, clear aligners have gradually gained the attention of orthodontists [1, 2]. Maxillary molar distalization is a strategy used when 2–3 mm of space is needed to achieve a class I relationship [3]. It can effectively alleviate anterior crowding, correct deep overjet and improve molar relationships. Molar distalization is often used for adults with class II malocclusion to gain clearance to improve the posterior occlusal relationship [4]. Orthodontists in clinical practice are often concerned about the efficiency of clear aligners when using them for maxillary molar distalization. The efficiency of maxillary molar distalization using clear aligners alone has been investigated in recent years [5, 6]. However, few studies have focused on maxillary molar distalization effects with clear aligners combined with intramaxillary and intermaxillary traction.

The traditionally used molar distalization appliances can be divided into extraoral (headgear compliance) or intraoral appliances (such as the pendulum appliance and the Jasper Jumper) [7]. Ravera et al. found that during molar distalization with clear aligners, the maximum stress of periodontal ligaments (PDLs) in maxillary anterior teeth was mainly distributed on the lingual side of central incisors and canines. This suggests a tendency toward labial inclination in the upper anterior teeth [4]. Therefore, reinforcing anterior anchorage when performing molar distalization with clear aligners is important. Anterior anchorage augmentation is primarily achieved using orthodontic mini-implants (OMIs) and/or class II elastics [8, 9]. Researchers have noted that OMIs implanted on the buccal side effectively decreased coronal-labial torque and alleviated stress in the anterior regions. However, limited research has been conducted on the mechanisms of OMIs implanted on the palatal side. Therefore, quantitative studies investigating the biomechanical impact of OMI implantation sites and traction force on anterior and posterior teeth are needed. The anchorage loss effect in mandibular dentition when using class II elastics also needs investigation.

The finite element method (FEM) is a numerical engineering technique that can be used to instantly calculate the initial tooth displacement after stress loading [10], and is widely used in biomechanical studies on clear aligners to evaluate the initial displacement and stress distribution of teeth after stress loading. FEM was demonstrated to be an effective tool for simulating orthodontic tooth movement patterns in recent years [11]. 

Therefore, the objective of the present study was to investigate the mechanism of maxillary molar distalization using clear aligners combined with intramaxillary and intermaxillary traction using the FEM. Clinical guidance was provided for the selection of intramaxillary and intermaxillary traction types in the process of maxillary molar distalization.

## Methods

One healthy adult female volunteer with class II malocclusion was selected as the subject. The study was approved by The West China Hospital of Stomatology Institutional Review Board (Ethical number: WCHS-IRB-CT-2021-260).

**Fig. 1:**
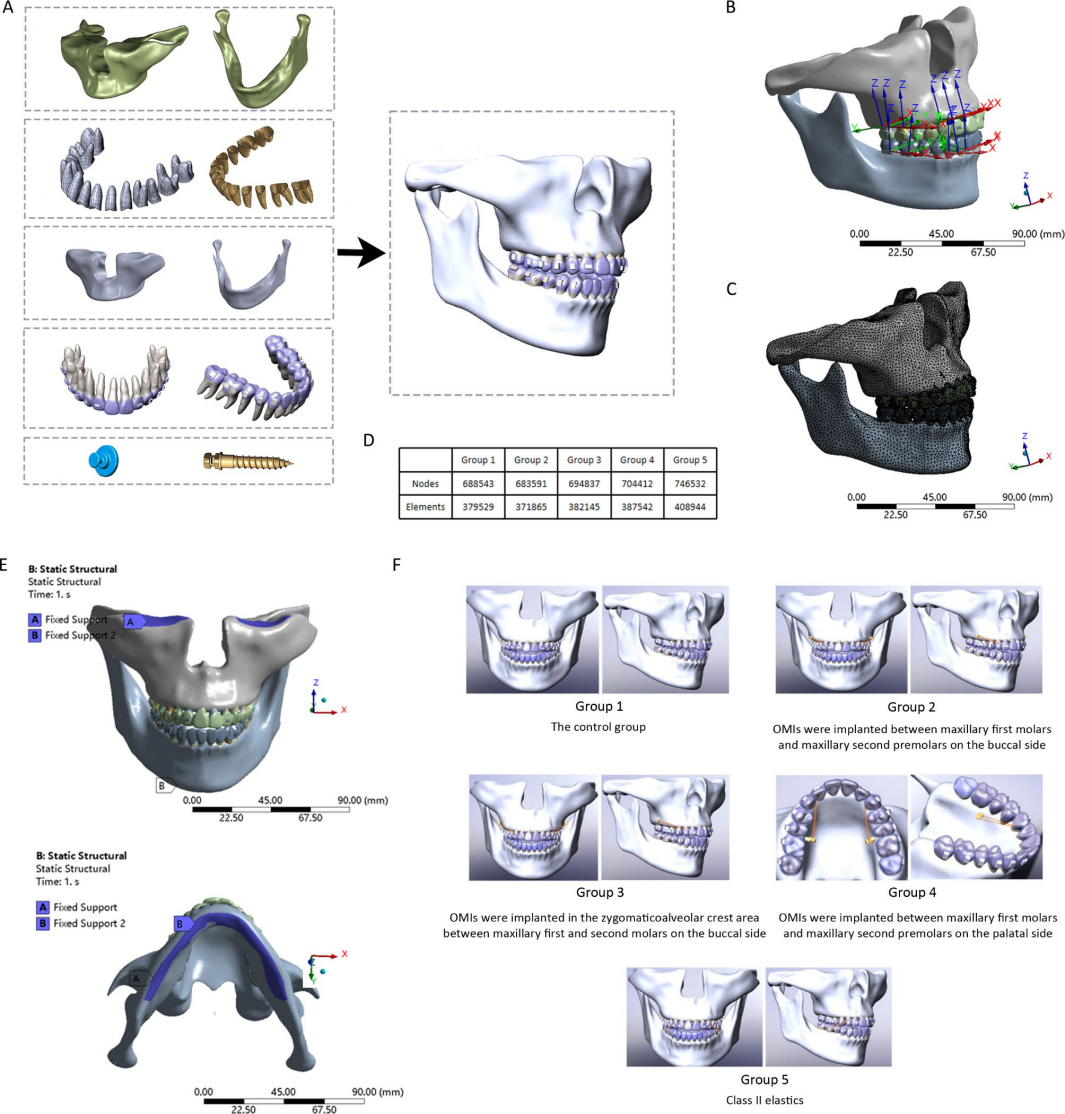
(**A**) Finite element models, consisting of maxillary and mandibular cortical bone, PDLs, maxillary and mandibular cancellous bone, maxillary and mandibular dentitions and clear aligners with attachments, and lingual buttons and OMIs (12 mm). (**B**) The global and local coordinate systems. (**C**) The mesh figure. (**D**) The nodes and elements. (**E**) The boundary conditions. Label A: the top of the maxilla was set as fixed support; Label B: movement of the nodes at the bottom edge of the mandible was constrained in all directions. (**F**) The sets of groups were designed on the basis of different intramaxillary and intermaxillary distraction modalities

Based on data obtained from cone beam computed tomography (CBCT), 3D models of the maxilla and mandible, as well as the maxillary and mandibular dentitions, were established using Mimics Research (version 20.0; Materialise, Belgium) and Geomagic Wrap 2017 (3D Systems, USA). According to the molar distalization protocol established by Angelalign (EA Medical Instruments, China), we set the initial maxillary molar distalization movements at 0.25 mm. The bilateral maxillary second molars were distally displaced by 0.25 mm along the X-axis of coordinate system. The coronal surface was evenly expanded by 0.75 mm outward, and the original coronal model was subtracted using Boolean operation. The edges of the clear aligner were trimmed, and precision cuts were made at the maxillary canine. Additionally, the position of the lingual button was reserved on the mesial buccal surfaces of mandibular first molars. A mandibular clear aligner was constructed in the same manner. The root surface was expanded by 0.25 mm outward to obtain the PDL of each tooth in the maxilla and mandible respectively. The maxilla and mandible were moved inward by 1.5 mm using the offset command in Geomagic and then cortical bone and cancellous bone models were established by Boolean subtraction operation instructions in Solidworks 2017 (Dassault Systemes, French). In accordance with Angelalign molar distalization protocol, the attachments were set as a vertical rectangular shape (3 mm high, 2 mm wide, and 1 mm thick), which were then combined with each tooth surface, with the intervening parts removed. The parameters of each attachment were evenly expanded by 0.75 mm outward and combined with the clear aligner model. The final clear aligner model was obtained after removing the intervening parts using Boolean operation. The bottom surface diameter of the lingual button model was set as 3.4 mm. OMI models were constructed with reference to the VectorTAS system (VectorTAS, USA), featuring OMIs with a length of 8 mm (diameter 1.4 mm) implanted between the maxillary first molars and second premolars, and OMIs with a length of 12 mm (diameter 2 mm) implanted in the infrazygomatic crest area. The assembly files of models were generated and imported into Ansys Workbench 19.0 (Ansy, USA) (Fig. 1A).

Material properties were assigned to each component (Table 1) [12]. Each material and tissue structure was set up as linear elastic. A local coordinate system was established for each tooth, with the origin located at the tooth centroid (Fig. 1B). The X-axis represents the mesiodistal displacement of teeth (positive values indicate mesial displacement), the Y-axis represents the labiolingual displacement (positive values indicate lingual displacemen), and the Z-axis represents the vertical displacement (positive values indicate intrusion). To quantify the displacement of each tooth, crown displacement nodes (the midpoints of incisal margins or cusps of anterior teeth, mesial buccal tips of the first molars), cervical displacement nodes (intersections of teeth body and buccal alveolar crest edges) and root displacement nodes (the apical points of anterior teeth, the mesial buccal root points of the first molars) were set. The contact conditions were set as follows: bonded relationship between cancellous bone and cortical bone, roots and PDLs, PDLs and cancellous/cortical bone, teeth and attachments; frictional relationship with a friction coefficient of 0.2 between teeth and clear aligners, as well as between attachments and clear aligners; and frictionless relationship between teeth. The distance between the maxillary and mandibular dentition was greater than the thickness of two aligners, ensuring that the maxillary and mandibular aligners was not in contact. The finite element mesh was divided by the discretization process (Fig. 1C), with the nodes and linear elements of each model illustrated in Fig. 1D. The boundary conditions were set as follows: the top of the maxilla was assigned a fixed support, while the movement of the nodes at the bottom edge of the mandible was constrained in all directions (Fig. 1E).

**Table 1:** Material properties

Component	Young’s modulus (MPa)	Poisson’s ratio
Cortical bone	13,700	0.30
Cancellous bone	1370	0.30
Teeth	19,600	0.30
PDL	0.67	0.45
Clear aligner	528	0.36
Attachment	12,500	0.36
Lingual button	206,000	0.30
OMI	103,400	0.35

PDL: Periodontal ligament; OMI: Orthodontic mini-implant

The OMI implantation and traction force loading were performed according to the design of each experimental group. Group 1: the control group; group 2: OMIs were implanted between the maxillary first molars and second premolars on the buccal side; group 3: OMIs were implanted in the infrazygomatic crest area between the maxillary first and second molars on the buccal side; group 4: OMIs were implanted between the maxillary first molars and second premolars on the palatal side; and group 5: class II elastics. OMIs were positioned 4 mm away from the alveolar crest. A force of 1.5 N was applied to both sides in experimental group 2–5 (Fig. 1F) [8, 13]. Only the results from the right side of the dentition were presented due to the similarity between the results of the left and right sides.

## Results

### Three-dimensional displacement of maxillary anterior teeth

**Fig. 2:**
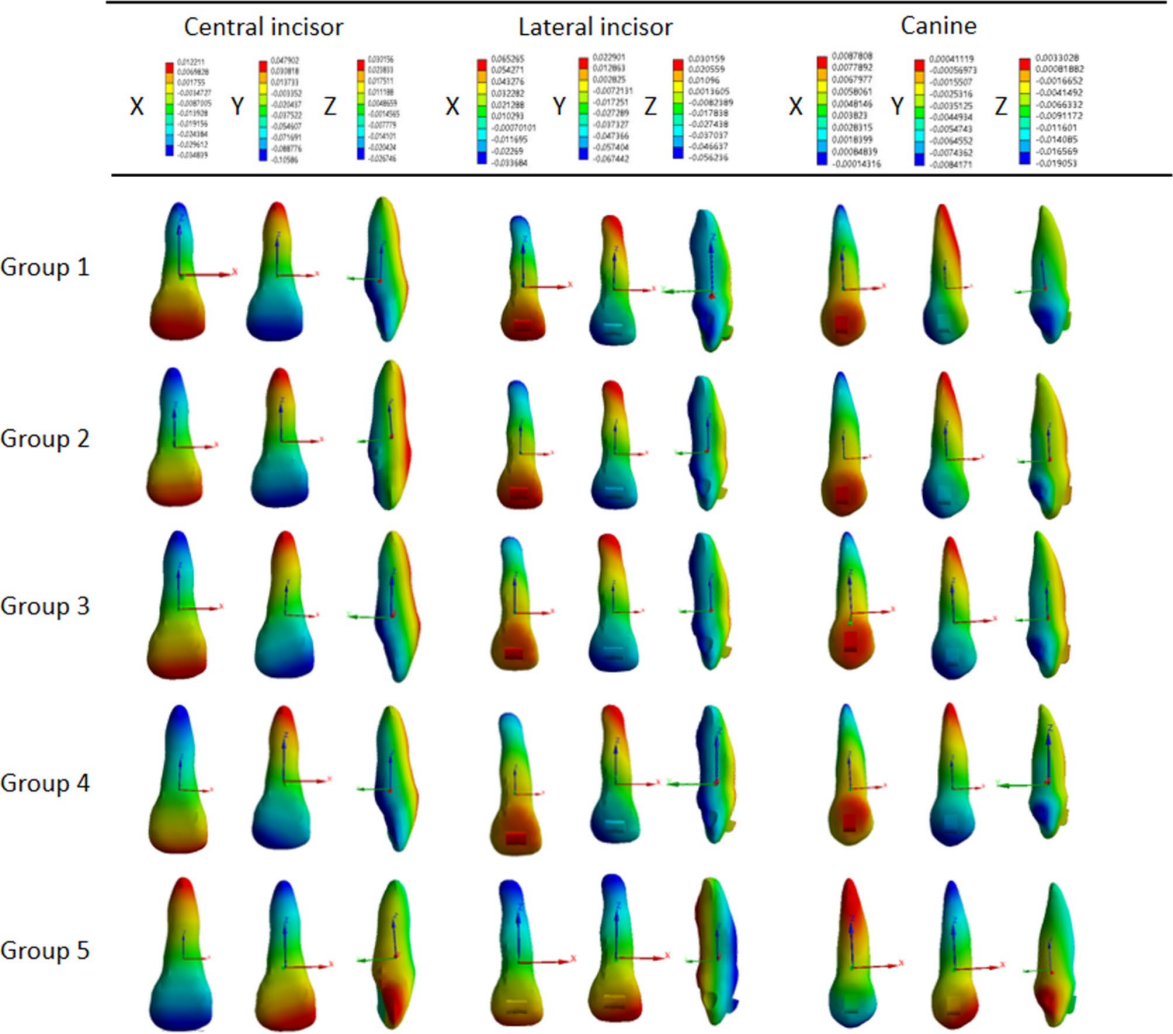
Three-dimensional displacement patterns of right maxillary anterior teeth. Group **1**: the control group; group **2**: OMIs were implanted between maxillary first molars and maxillary second premolars on the buccal side; group **3**: OMIs were implanted in the infrazygomatic crest area between maxillary first and second molars on the buccal side; group **4**: OMIs were implanted between maxillary first molars and maxillary second premolars on the palatal side; and group **5**: class II elastics

The right maxillary anterior teeth showed mesial labial inclination with intrusion in all the OMI groups. In contrast, the maxillary anterior teeth in the class II elastics group exhibited distal lingual inclination with extrusion (Fig. 2).

**Fig. 3:**
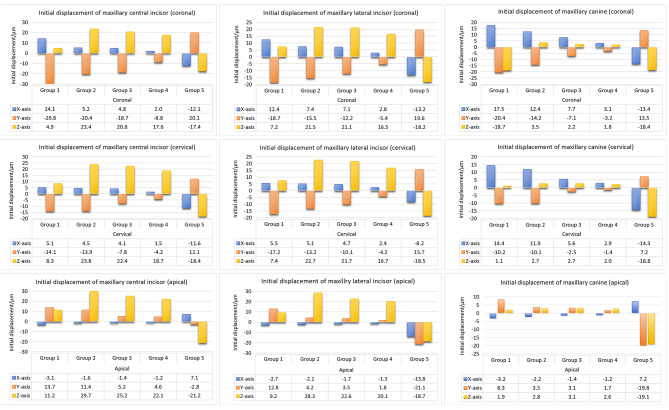
Initial displacement of maxillary anterior teeth in mesiodistal (X-axis), labiolingual (Y-axis), and vertical (Z-axis) directions in five groups (µm). Group **1**: the control group; group **2**: OMIs were implanted between maxillary first molars and maxillary second premolars on the buccal side; group **3**: OMIs were implanted in the infrazygomatic crest area between maxillary first and second molars on the buccal side; group **4**: OMIs were implanted between maxillary first molars and maxillary second premolars on the palatal side; and group **5**: class II elastics. X-axis: Positive values indicate a mesial inclination tendency, and negative values indicate a distal inclination tendency; Y-axis: positive values indicate a palatal inclination tendency, and negative values indicate a buccal inclination tendency; Z-axis: positive values indicate an intrusion tendency, and negative values indicate an elongation tendency

The greatest intrusion of the upper anterior teeth occurred when OMIs were placed between the maxillary first molars and second premolars on the buccal side. This was followed by group 3, in which OMIs were implanted between the maxillary first and second molars on the buccal side. The intrusion was smaller when OMIs were implanted on the palatal side, but it was still higher than that in the control group (Fig. 3). The coronal labial inclination of group 2 and 3, with OMIs implanted on the buccal side, was lower than that of the control group. The labial inclination of crowns in group 4, with OMIs implanted on the palatal side, was the lowest (Fig. 3). The mesial movement of the crowns of the anterior teeth in group 2 and 3, with OMIs implanted on the buccal side, was significantly lower than that of the control group. Additionally, the crowns in group 4, in which OMIs were implanted on the palatal side, showed the lowest mesial movement (Fig. 3).

### Three-dimensional displacement of maxillary molars

**Fig. 4:**
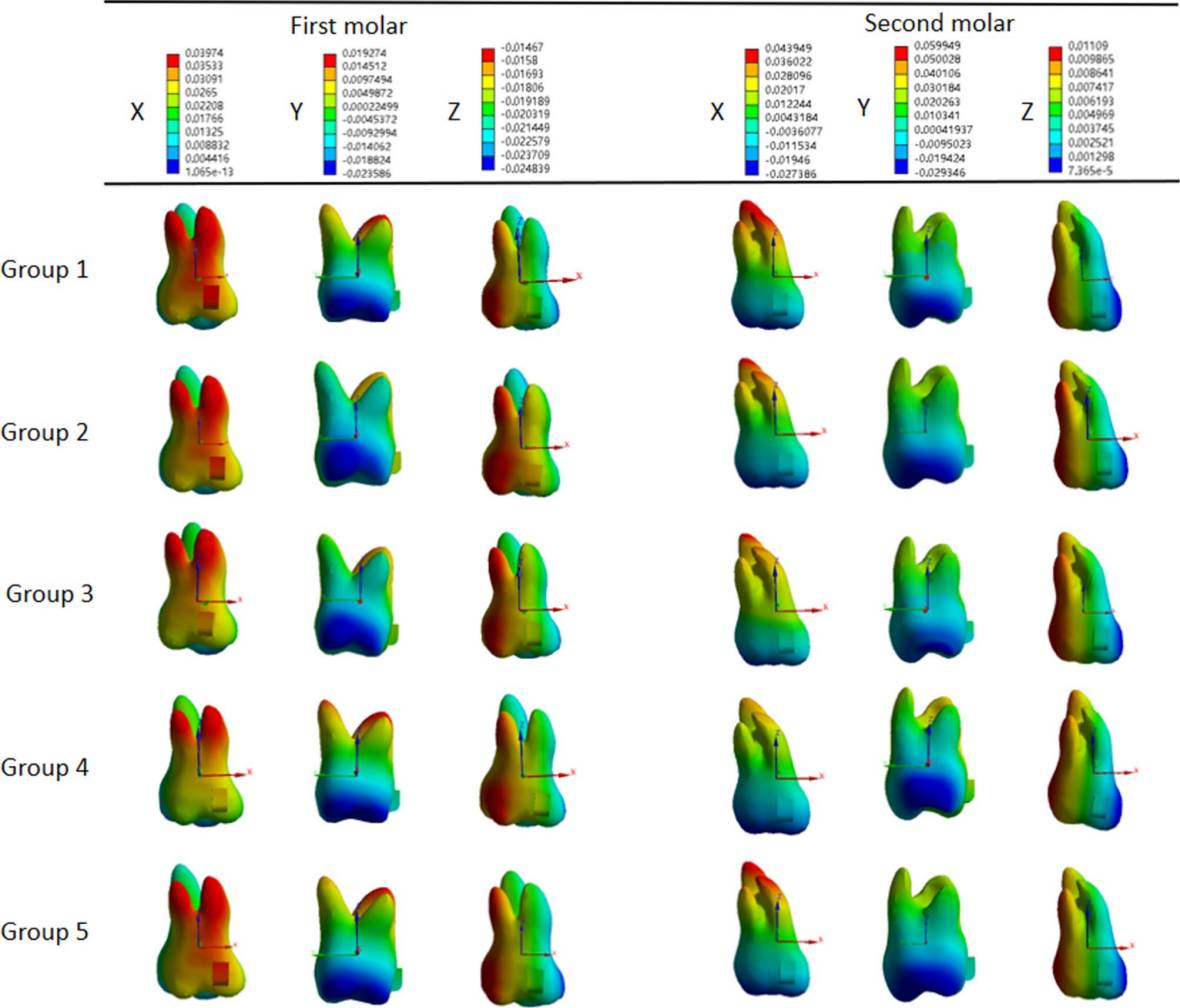
Three-dimensional displacement patterns of right maxillary molars. Group **1**: the control group; group **2**: OMIs were implanted between maxillary first molars and maxillary second premolars on the buccal side; group **3**: OMIs were implanted in the infrazygomatic crest area between maxillary first and second molars on the buccal side; group **4**: OMIs were implanted between maxillary first molars and maxillary second premolars on the palatal side; and group **5**: class II elastics

The right maxillary first molars mainly exhibited mesial buccal inclination with extrusion and the right maxillary second molars exhibited distal buccal inclination with intrusion in all groups (Fig. 4).

**Fig. 5:**
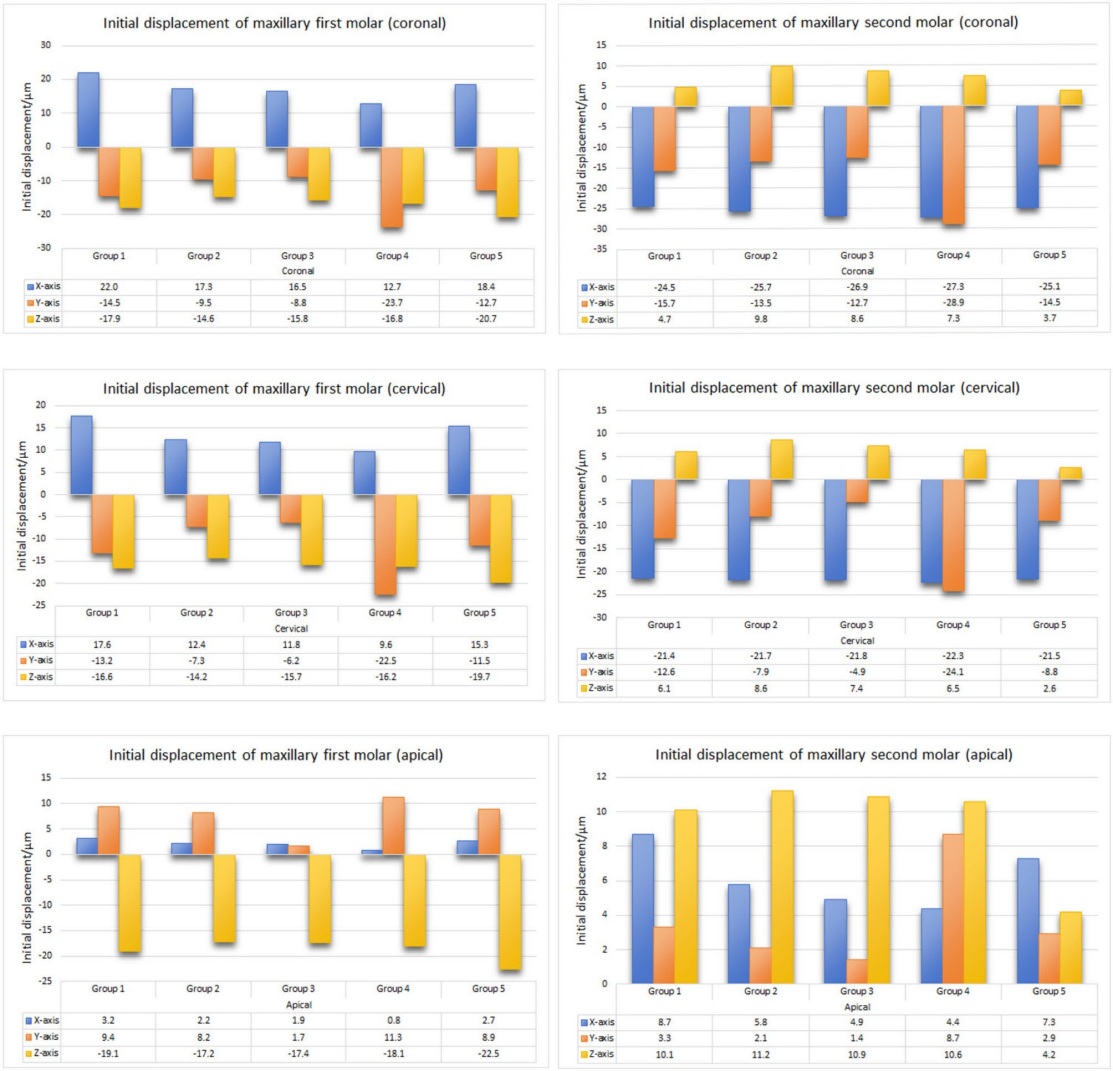
Initial displacement of maxillary molars in mesiodistal (X-axis), labiolingual (Y-axis), and vertical (Z-axis) directions in five groups (µm). Group **1**: the control group; group **2**: OMIs were implanted between maxillary first molars and maxillary second premolars on the buccal side; group **3**: OMIs were implanted in the infrazygomatic crest area between maxillary first and second molars on the buccal side; group **4**: OMIs were implanted between maxillary first molars and maxillary second premolars on the palatal side; and group **5**: class II elastics. X-axis: Positive values indicate a mesial inclination tendency, and negative values indicate a distal inclination tendency; Y-axis: positive values indicate a palatal inclination tendency, and negative values indicate a buccal inclination tendency; Z-axis: positive values indicate an intrusion tendency, and negative values indicate an elongation tendency

The extrusion amount of the maxillary first molars was lower in group 2, 3, and 4, in which OMIs were implanted on the buccal or palatal side, compared to the control group. Conversely, the intrusion amount of the maxillary second molars was higher in groups 2, 3, and 4 compared to the control group. Among them, the highest intrusion value was observed in group 2, while the lowest was observed in the palatal traction group (Fig. 5). The buccal inclination of crowns in groups with OMIs implanted on the buccal side or class II elastics was lower than that of the control group, while it was the highest in the palatal traction group (Fig. 5). Distal displacement of the second molar was the lowest in the control group and highest in the palatal traction group (Fig. 5).

### Three-dimensional displacement of mandibular anterior teeth and molars in the Class II elastics group

**Fig. 6:**
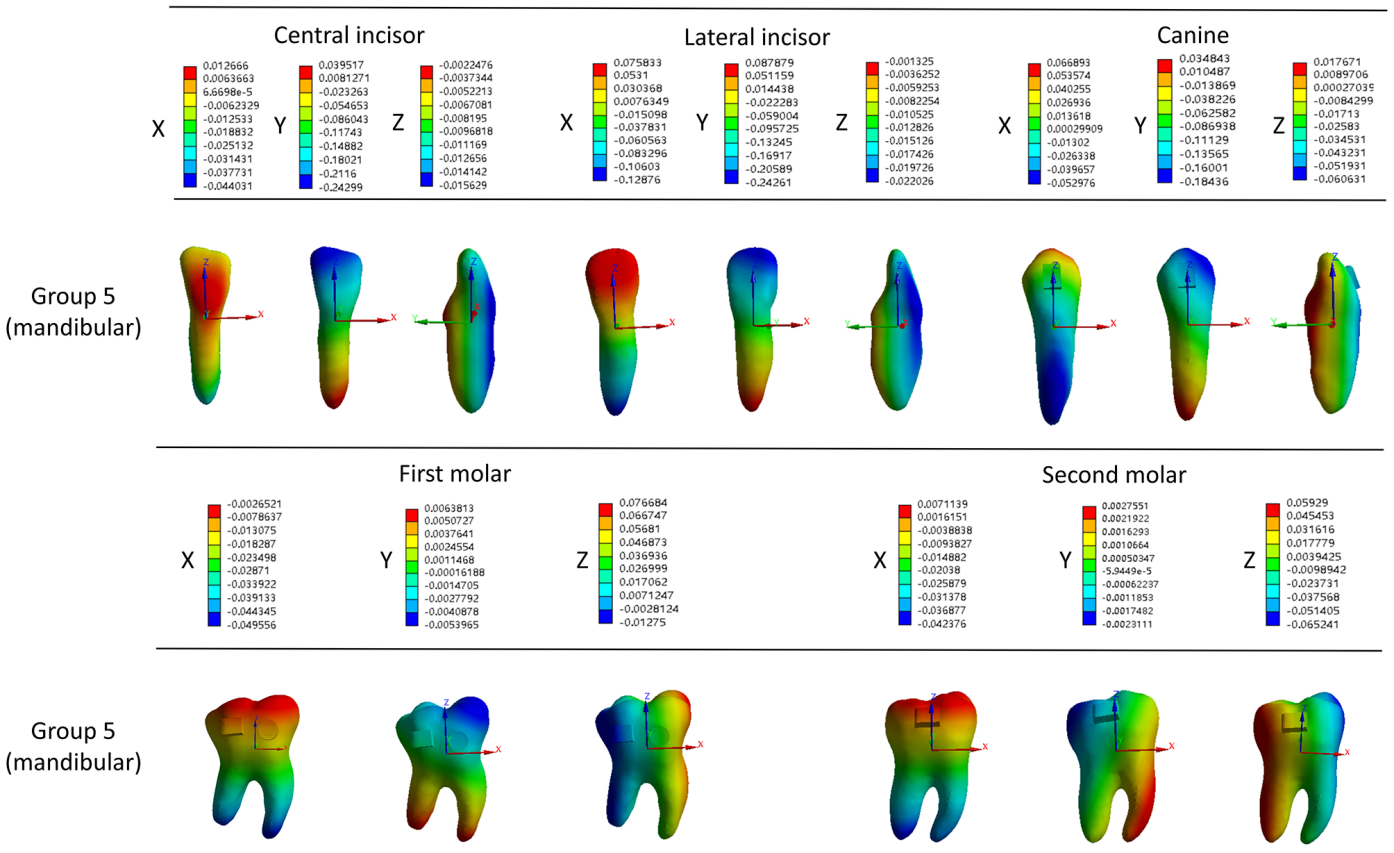
Three-dimensional displacement patterns of right mandibular anterior teeth and molars in class II elastics group

In the class II elastics group, the mandibular anterior teeth exhibited mesial labial inclination with intrusion, and the mandibular first molars exhibited mesial buccal inclination with extrusion (Fig. 6).

### Stress distribution and maximum von mises stress of maxillary anterior teeth and molars PDLs

**Fig. 7:**
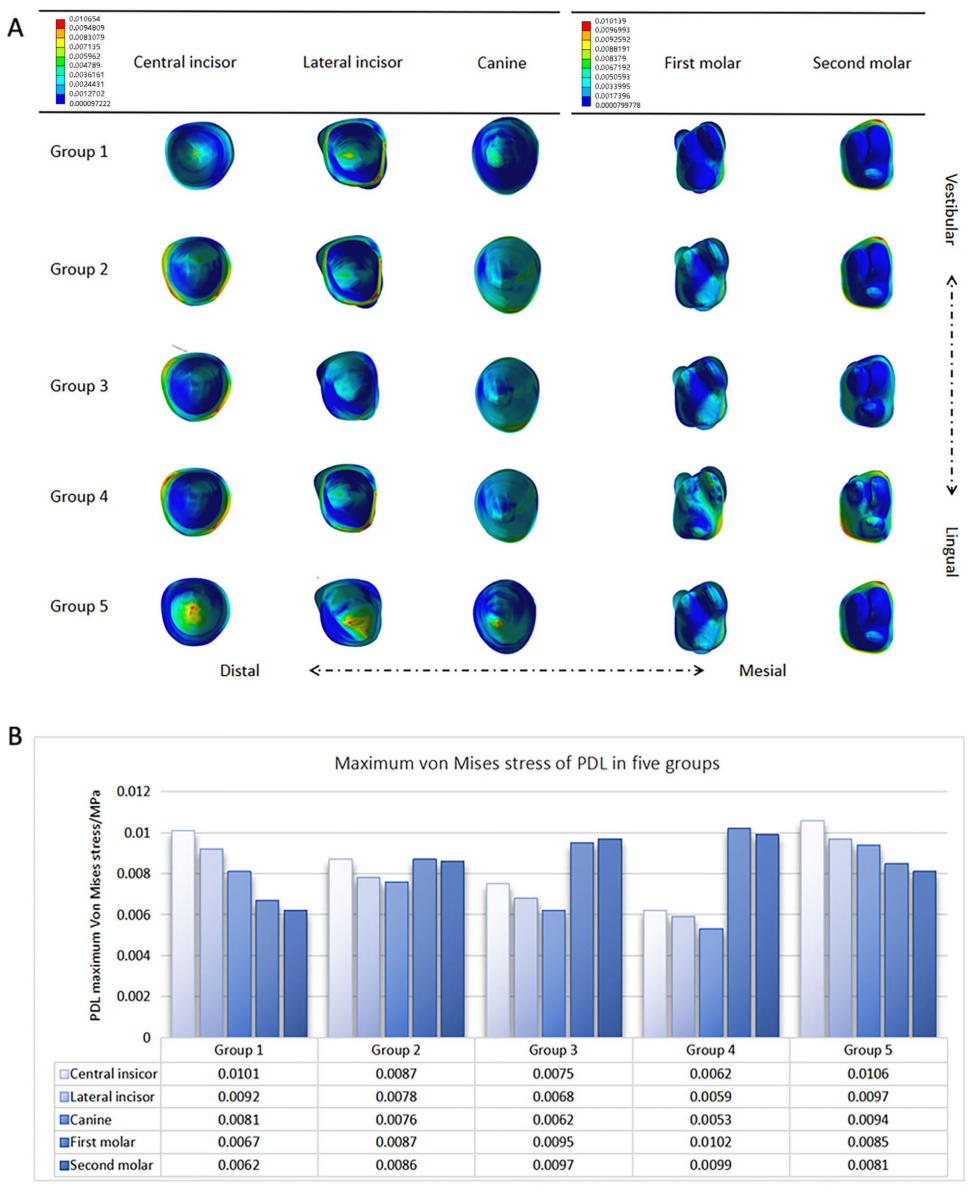
(**A**) Stress distribution around PDLs of right maxillary anterior teeth and molars. (**B**) Maximum von Mises stress of PDL in five groups (MPa). Group **1**: the control group; group **2**: OMIs were implanted between maxillary first molars and maxillary second premolars on the buccal side; group **3**: OMIs were implanted in the infrazygomatic crest area between maxillary first and second molars on the buccal side; group **4**: OMIs were implanted between maxillary first molars and maxillary second premolars on the palatal side; and group **5**: class II elsastics

Stress distribution around PDLs is positively correlated with root resorption, which can help predict the likelihood of root resorption [14]. The maximum von Mises stress of PDLs can reflect the maximum equivalent stress under various force application conditions. The stress distribution analysis in the anterior teeth showed that both the control and class II elastic groups had significant stress concentrations at the apical position. However, the stress concentrations in the three groups with OMIs implanted were less significant than those observed in the two aforementioned groups. The stress distribution analysis in molars showed that the concentrations were more pronounced in the three groups with OMIs and the group using class II elastics compared to the control group. This was particularly evident in the palatal traction group, where stress concentration was observed on the mesial side of the first molar and the distal side of the second molar (Fig. 7A). The maximum von Mises stress was the highest in the class II elastics group in the anterior region, while it was the highest in the palatal traction group in the posterior region (Fig. 7B).

## Discussion

In this experiment, a 3D finite element model of clear aligners combined with intramaxillary and intermaxillary traction in molar distalization was established to simulate the initial displacement of the target teeth. The maximum von Mises stress of PDLs in each traction method was determined to examine the effect of different types of intramaxillary and intermaxillary traction, providing theoretical guidance for the clinical use of each type of traction.

The maxillary anterior teeth in each group exhibited different degrees of intrusion and labial inclination, except for the class II elastics group. This indicates an anchorage loss of the maxillary anterior teeth when OMIs were used for maxillary intramaxillary traction. Among the maxillary anterior teeth, anchorage loss was mainly characterized by sagittal anchorage loss. In the four experimental groups, the palatal traction group exhibited the best torque control and the least amount of intrusion for anterior teeth. This could be attributed to the larger sagittal component of the traction force compared to the other experimental groups, as well as the smaller vertical component. The distalization efficiency in the posterior region was primarily evaluated by measuring the mesiodistal displacement of the maxillary second molars. The palatal traction group demonstrated the highest efficiency in molar distalization; however, there was a potential risk of buccal anchorage loss in the posterior region. Furthermore, the displacement of the two buccal traction groups demonstrated that the molar distalization and anterior anchorage protection efficiency increased as the OMIs were implanted between the first and second molars. However, in the clinical implantation of OMIs, the influence of factors such as the distance between the root of maxillary molars and the floor of maxillary sinus, root morphology, and intraosseous root volume must be examined [15, 16]. Scholars have reported that the two sites for OMIs placement with high success rates are the palatal side and the infrazygomatic crest [17]. The safety and stability of palatal OMIs implantation between the maxillary first molars and second premolars can be attributed to the wide interradicular space, which prevents contact between the OMIs and the roots, as well as the thick palatal cortical bone [18]. It has been noted that OMIs implanted in the infrazygomatic crest area can achieve en-masse distalization of posterior teeth without repositioning [19]. Rosa et al. included a sample of 25 patients in their study. When OMIs were implanted in the infrazygomatic crest area approximately 11 mm above the occlusal plane, they achieved 4 mm of the maxillary first molar distalization without observed failure of the OMIs [20]. 

The efficiency of molar distalization using class II elastics was not as good as that of OMIs, and risks, such as bone fenestration and bone dehiscence in the mandibular anterior region, should be minimized when performing maxillary molar distalization with class II elastics [21, 22]. In addition, molar extrusion may also result in an inclination of the occlusal plane or an increase in the depth of the Spee curve.

Shibata et al. reported that when the local PDL fluid static pressure was higher than the capillary pressure in this area, the capillaries would be compressed, leading to adverse effects, such as periodontal tissue necrosis and root resorption. In this experiment, the maximum von Mises stress of the PDLs in each group did not exceed their upper limit [23]. This indicates that intramaxillary and intermaxillary traction with a force of 1.5 N was considered safe. The analysis of stress distribution around the PDLs showed that the highest stress concentration was observed in the class II elastic group in the anterior teeth, while the palatal implant group exhibited the highest stress concentration in molars. The analysis of the maximum von Mises stress of the PDLs showed that as the implant position was moved more posteriorly, the load on the anterior teeth decreased, and the load on the posterior teeth increased. Palatal traction reduced the anterior PDL load and increased the molar PDL load compared to buccal traction.

A limitation of this study is that the efficacy of appliance in practice is lower than predicted due to the processing technologies and material properties of clear aligners [24–26]. In addition, finite element studies are static analyses that only demonstrate an initial displacement, which is independent of the dynamic tooth movement process observed clinically. Several factors need to be considered when determining whether displacement can be used to evaluate the final movement effect, such as patient compliance, occlusal force loading, the remodeling of alveolar bones, and periodontal tissue health [27, 28]. Finite element modeling in this study was based on the CBCT of a single patient, limiting the ability to generalize simulation inferences to a larger population. Considering the variability in bone density, anatomical structures, and cortical bone thickness, the conclusions of this study should be interpreted with caution. To enhance the validity of these findings, further clinical studies and case reports, incorporating larger sample sizes, are necessary. More research is needed to analyze 3D dentition displacement and PDL stress distribution when the magnitude of the traction force, rather than the direction, changes. Whether an ideal ratio between the sequential distalization versus the activating force from OMIs exists also needs to be investigated [29, 30]. 

## Conclusions

1. OMIs implanted on the palatal side prevented the labial inclination of anterior teeth and enhanced the efficiency of molar distalization compared to those implanted on the buccal side.
2. OMIs implanted in the infrazygomatic crest area between the first and second molars on the buccal side protected the anterior anchorage and enhanced the molar distalization effect compared to OMIs implanted between the first molars and the second premolars on the buccal side.
3. The effect of class II elastics in molar distalization was less significant than that of OMIs, with potential adverse effects, including the extrusion and lingual inclination of the upper anterior teeth, as well as mandibular anchorage loss.

## Data Availability

Not applicable.
